# Supplementary material for: Moderate and Stable Pain Reductions as a Result of Interdisciplinary Pain Rehabilitation—A Cohort Study from the Swedish Quality Registry for Pain Rehabilitation (SQRP)
Source: J Clin Med. 2019 Jun 24;8(6):905. doi: 10.3390/jcm8060905 (PMC6617026; doi:10.3390/jcm8060905)
Supplement: Supplementary file 1 [file jcm-08-00905-s001.pdf]

Supplementary Table S1. Pre-IMMRP situation for patients reporting their outcomes at 12-m FU and those not reporting their outcomes at 12-m FU.

Variables	Report at 12-m FU	n:8096–8904	No report at 12-m FU	n:6069-6808
Women (%)	77.1		74.6	
Outside-Europe (%)	9.3		12.8	
University (%)	25.5		24.8	
	Mean	SD	Mean	SD
Age (years)	43.64	10.81	42.79	10.64
Days no work	1106[a]	2540	1006[c]	2320
Pain duration	3086	3429	2984	3195
Persistent pain duration	2393[b]	3168	2300[d]	2661
PRI	14.29	8.27	14.09	8.55
NRS-7days	6.83	1.71	6.91	1.74
HADS-A	8.73	4.69	9.37	4.83
HADS-D	8.19	4.37	8.86	4.50
MPI-Pain-severity	4.36	0.91	4.43	0.96
MPI-Pain-interfer	4.34	1.02	4.43	1.04
MPI-LifeCon	2.78	1.10	2.65	1.11
MPI-Distress	3.42	1.27	3.52	1.27
MPI-Socsupp	4.17	1.33	4.15	1.37
MPI-punish	1.70	1.35	1.79	1.40
MPI-protect	2.93	1.39	2.99	1.42
MPI-distract	2.51	1.18	2.57	1.22
MPI -GAI	2.47	0.83	2.39	0.85
EQ-5D-index	0.27	0.31	0.24	0.30
EQ-VAS	41.76	19.29	40.57	19.18
sf36-pf	53.03	20.29	52.07	21.07
sf36-rp	13.08	24.96	11.80	23.69
sf36-bp	24.59	14.06	23.93	15.03
sf36-gh	42.54	20.47	40.34	20.14
sf36-vt	24.94	18.78	22.87	18.23
sf36-sf	48.92	25.46	45.11	24.82
sf36-re	44.58	43.18	39.88	42.41
sf36-mh	56.30	21.13	53.20	21.67

[a]:n=3125, [b]:n=6779, [c]:n=2694, [d]:n=5060

NRS-7days = Pain intensity as measured by a numeric rating scale for the previous 7 days; HADS = Hospital Anxiety and Depression Scale; MPI = Multidimensional Pain Inventory; EQ-5D-index = The index of the European quality of life instrument; EQ-VAS = The European quality of life instrument thermometer-like scale; sf36 = The Short Form (36) Health Survey; PRI = Pain Region Index. For explanations of the subscale abbreviations see Methods.

Supplementary Table S2. OPLS regressions of MIS at post IMMRP in the three clusters; variables in bold type are significant

**Cluster 1**			**Cluster 2**			**Cluster 3**		
Variables pre IMMRP	VIP	p(corr)	Variables pre IMMRP	VIP	p(corr)	Variables pre IMMRP	VIP	p(corr)
**sf36-mh**	**1.59**	**-0.73**	**sf36-mh**	**1.84**	**-0.84**	**sf36-mh**	**1.54**	**-0.75**
**MPI-Distress**	**1.53**	**0.70**	**MPI-Distress**	**1.79**	**0.81**	**HADS-D**	**1.54**	**0.75**
**MPI-Pain-interfer**	**1.50**	**0.69**	**HADS-D**	**1.70**	**0.76**	**MPI-Pain-interfer**	**1.51**	**0.73**
**HADS-D**	**1.48**	**0.68**	**HADS-A**	**1.63**	**0.73**	**sf36-sf**	**1.48**	**-0.72**
**MPI-LifeCon**	**1.48**	**-0.68**	**sf36-sf**	**1.55**	**-0.71**	**MPI-Distress**	**1.47**	**0.72**
**sf36-sf**	**1.47**	**-0.67**	**MPI-LifeCon**	**1.54**	**-0.69**	**MPI-LifeCon**	**1.41**	**-0.68**
**EQ-5D-index**	**1.41**	**-0.65**	**sf36-re**	**1.41**	**-0.65**	**sf36-vt**	**1.31**	**-0.64**
**HADS-A**	**1.34**	**0.62**	**MPI-Pain-interfer**	**1.35**	**0.61**	**sf36-bp**	**1.30**	**-0.64**
**sf36-bp**	**1.34**	**-0.62**	**sf36-vt**	**1.32**	**-0.60**	**EQ-5D-index**	**1.29**	**-0.63**
**MPI-Pain-severity**	**1.33**	**0.61**	**EQ-5D-index**	**1.17**	**-0.54**	**HADS-A**	**1.28**	**0.62**
**EQ-VAS**	**1.25**	**-0.58**	**sf36-bp**	**1.03**	**-0.47**	**MPI-Pain-severity**	**1.24**	**0.60**
**sf36-vt**	**1.18**	**-0.54**	**MPI-Pain-severity**	**1.01**	**0.45**	**EQ-VAS**	**1.20**	**-0.58**
**sf36-re**	**1.09**	**-0.50**	EQ-VAS	1.00	-0.47	**sf36-gh**	**1.19**	**-0.58**
**sf36-gh**	**1.09**	**-0.50**	sf36-gh	0.99	-0.46	**sf36-re**	**1.19**	**-0.58**
**NRS-7days**	**1.05**	**0.49**	sf36-rp	0.83	-0.38	**sf36-pf**	**1.08**	**-0.52**
MPI-GAI	0.90	-0.41	NRS-7days	0.77	0.35	**NRS-7days**	**1.07**	**0.52**
sf36-pf	0.90	-0.42	MPI-GAI	0.66	-0.30	sf36-rp	0.93	-0.45
sf36-rp	0.75	-0.34	sf36-pf	0.55	-0.25	MPI-GAI	0.82	-0.40
MPI-punish	0.48	0.22	MPI-punish	0.54	0.26	MPI-punish	0.61	0.29
PRI	0.45	0.21	Age	0.43	-0.19	PRI	0.58	0.28
Outside-Europe	0.43	0.20	Outside-Europe	0.40	0.18	Outside-Europe	0.34	0.16
MPI-protect	0.35	0.16	Pain duration	0.26	-0.13	MPI-protect	0.22	0.10
MPI-Socsupp	0.27	0.12	Persistent Pain duration	0.25	-0.15	MPI-distract	0.19	0.09
MPI-distract	0.26	0.12	MPI-distract	0.20	0.10	University	0.18	-0.09
Age	0.25	-0.11	MPI-protect	0.19	0.09	Pain duration	0.15	0.07
University	0.18	-0.09	PRI	0.19	0.08	MPI-Socsupp	0.14	0.07
Pain duration	0.14	-0.07	University	0.07	0.03	Persistent Pain duration	0.12	0.06
Gender	0.06	-0.03	MPI-Socsupp	0.05	0.03	Gender	0.05	0.03
Days no work	0.05	-0.02	Days no work	0.05	-0.06	Age	0.04	0.02
Persistent Pain duration	0.04	-0.03	Gender	0.00	0.00	Days no work	0.01	0.01
R^2	0.06		R^2	0.01		R^2	0.04	
Q^2	0.05		Q^2	0.01		Q^2	0.04	
n	2205		n	7932		n	4523	
CV ANOVA	<0.001		CV ANOVA	<0.001		CV ANOVA	<0.001	

VIP (VIP>1.0 is significant) and p(corr) are reported for each regressor. The sign of p(corr) indicates the direction of the correlation with the dependent variable (+ = positive correlation; - = negative correlation). The four bottom rows of each regression report R^2, Q^2, and *P*-value of the CV-ANOVA, and number of patients included in the regression (n). NRS-7days = Pain intensity as measured by a numeric rating scale for the previous 7 days; HADS = Hospital Anxiety and Depression Scale; MPI = Multidimensional Pain Inventory; EQ-5D-index = The index of the European quality of life instrument; EQ-VAS = The European quality of life instrument thermometer-like scale; sf36 = The Short Form (36) Health Survey; PRI = Pain Region Index. For explanations of the subscale abbreviations see Methods.

Supplementary Table S3. OPLS regressions of MIS at 12-month FU in the three clusters; variables in bold type are significant.

**Cluster 1**			**Cluster 2**			**Cluster 3**		
Variables pre IMMRP	VIP	p(corr)	Variables pre IMMRP	VIP	p(corr)	Variables pre IMMRP	VIP	p(corr)
**MPI-Pain-interfer**	**1.58**	**0.73**	**MPI-Pain-severity**	**1.75**	**-0.61**	**sf36-mh**	**1.57**	**-0.78**
**sf36-mh**	**1.52**	**-0.70**	**sf36-pf**	**1.71**	**0.59**	**HADS-D**	**1.55**	**0.77**
**MPI-Distress**	**1.52**	**0.70**	**NRS-7days**	**1.60**	**-0.56**	**MPI-Distress**	**1.52**	**0.76**
**sf36-sf**	**1.52**	**-0.70**	**sf36-bp**	**1.51**	**0.52**	**MPI-Pain-interfer**	**1.47**	**0.73**
**MPI-LifeCon**	**1.47**	**-0.68**	**Persistent Pain duration**	**1.46**	**-0.47**	**sf36-sf**	**1.46**	**-0.73**
**EQ-5D-index**	**1.44**	**-0.66**	**MPI-Pain-interfer**	**1.41**	**-0.49**	**MPI-LifeCon**	**1.45**	**-0.72**
**MPI-Pain-severity**	**1.40**	**0.64**	**PRI**	**1.34**	**-0.46**	**HADS-A**	**1.35**	**0.67**
**HADS-D**	**1.39**	**0.64**	**MPI-protect**	**1.27**	**-0.44**	**sf36-bp**	**1.28**	**-0.64**
**sf36-bp**	**1.38**	**-0.64**	**sf36-gh**	**1.21**	**0.42**	**EQ-5D-index**	**1.27**	**-0.63**
**HADS-A**	**1.29**	**0.60**	**EQ-5D-index**	**1.17**	**0.40**	**sf36-vt**	**1.26**	**-0.62**
**sf36-vt**	**1.19**	**-0.55**	**EQ-VAS**	**1.14**	**0.39**	**MPI-Pain-severity**	**1.24**	**0.62**
**EQ-VAS**	**1.17**	**-0.54**	**Pain duration**	**1.13**	**-0.39**	**EQ-VAS**	**1.18**	**-0.59**
**NRS-7days**	**1.15**	**0.53**	**Days no work**	**1.03**	**-0.38**	**sf36-re**	**1.18**	**-0.59**
sf36-re	1.00	-0.46	**MPI-distract**	**1.02**	**-0.35**	**sf36-gh**	**1.17**	**-0.58**
sf36-gh	0.98	-0.45	MPI-Socsupp	0.98	-0.34	**NRS-7days**	**1.04**	**0.52**
sf36-pf	0.93	-0.43	sf36-vt	0.78	0.27	**sf36-pf**	**1.01**	**-0.51**
MPI-GAI	0.85	-0.39	University	0.77	0.27	sf36-rp	0.92	-0.46
sf36-rp	0.71	-0.32	sf36-sf	0.75	0.26	MPI-GAI	0.88	-0.44
PRI	0.58	0.27	sf36-rp	0.71	0.25	MPI-punish	0.72	0.35
Outside-Europe	0.51	0.24	Outside-Europe	0.66	-0.23	PRI	0.54	0.27
MPI-protect	0.48	0.22	Age	0.55	-0.19	Outside-Europe	0.37	0.19
MPI-distract	0.40	0.19	MPI-GAI	0.51	0.18	University	0.18	-0.09
MPI-punish	0.39	0.18	MPI-LifeCon	0.42	0.15	MPI-protect	0.17	0.09
MPI-Socsupp	0.29	0.14	HADS-D	0.40	-0.14	days-no-work	0.11	0.06
Age	0.27	-0.13	MPI-Distress	0.23	-0.08	MPI-distract	0.09	0.05
Days no work	0.14	0.06	HADS-A	0.13	-0.05	Gender	0.06	-0.03
Pain duration	0.12	-0.05	MPI-punish	0.10	0.03	MPI-Socsupp	0.05	0.03
Gender	0.09	-0.04	Gender	0.09	0.03	Age	0.05	-0.02
University	0.09	-0.04	sf36-re	0.08	-0.02	Persistent Pain duration	0.02	-0.01
Persist Pain duration	0.07	-0.03	sf36-mh	0.05	0.02	Pain duration	0.01	0.01
R^2	0.07		R^2	0.02		R^2	0.05	
Q^2	0.07		Q^2	0.01		Q^2	0.05	
n	1099		n	4123		n	3629	
CV ANOVA	<0.001		CV ANOVA	<0.001		CV ANOVA	<0.001	

VIP (VIP>1.0 is significant) and p(corr) are reported for each regressor. The sign of p(corr) indicates the direction of the correlation with the dependent variable (+ = positive correlation; - = negative correlation). The four bottom rows of each regression report R^2, Q^2, and *P*-value of the CV-ANOVA, and number of patients included in the regression (n). NRS-7days = Pain intensity as measured by a numeric rating scale for the previous 7 days; HADS = Hospital Anxiety and Depression Scale; MPI = Multidimensional Pain Inventory; EQ-5D-index = The index of the European quality of life instrument; EQ-VAS = The European quality of life instrument thermometer-like scale; sf36 = The Short Form (36) Health Survey; PRI = Pain Region Index. For explanations of the subscale abbreviations see Methods.
